#  Association of Surgical Treatment With Adverse Events and Mortality Among Medicare Beneficiaries With Proximal Humerus Fracture

**DOI:** 10.1001/jamanetworkopen.2019.18663

**Published:** 2020-01-10

**Authors:** Sarah B. Floyd, Charles Thigpen, Michael Kissenberth, John M. Brooks

**Affiliations:** 1 Center for Effectiveness Research in Orthopaedics, University of South Carolina, Greenville; 2 Department of Health Services Policy and Management, Arnold School of Public Health, University of South Carolina, Columbia; 3 ATI Physical Therapy, Greenville, South Carolina; 4 Steadman Hawkins Clinic of the Carolinas, Prisma Health System, Greenville, South Carolina

## Abstract

**Importance:**

Meta-analyses of randomized clinical trials suggest that the advantages and risks of surgery compared with conservative management as the initial treatment for proximal humerus fracture (PHF) vary, or are heterogeneous across patients. Substantial geographic variation in surgery rates for PHF suggests that the optimal rate of surgery across the population of patients with PHF is unknown.

**Objective:**

To use geographic variation in treatment rates to assess the outcomes associated with higher rates of surgery for patients with PHF.

**Design, Setting, and Participants:**

This comparative effectiveness research study analyzed all fee-for-service Medicare beneficiaries with proximal humerus fracture in 2011 who were continuously enrolled in Medicare Parts A and B for the 365-day period before and immediately after their index fracture. Data analysis was performed January through June 2019.

**Exposure:**

Undergoing 1 of the commonly used surgical procedures in the 60 days after an index fracture diagnosis.

**Main Outcomes and Measures:**

Risk-adjusted area surgery ratios were created for each hospital referral region as a measure of local area practice styles. Instrumental variable approaches were used to assess the association between higher surgery rates and adverse events, mortality risk, and cost at 1 year from Medicare’s perspective for patients with PHF in 2011. Instrumental variable models were stratified by age, comorbidities, and frailty. Instrumental variable estimates were compared with estimates from risk-adjusted regression models.

**Results:**

The final cohort included 72 823 patients (mean [SD] age, 80.0 [7.9] years; 13 958 [19.2%] men). The proportion of patients treated surgically ranged from 1.8% to 33.3% across hospital referral regions in the United States. Compared with conservatively managed patients, surgical patients were younger (mean [SD] age, 80.4 [8.1] years vs 78.0 [7.2] years; *P* < .001) and healthier (Charlson Comorbidity Index score of 0, 14 863 [24.4%] patients vs 3468 [29.1%] patients; Function-Related Indicator score of 0, 20 720 [34.0%] patients vs 4980 [41.8%] patients; *P* < .001 for both), and a larger proportion were women (49 030 [80.5%] patients vs 9835 [82.5%] patients; *P* < .001). Instrumental variable analysis showed that higher rates of surgery were associated with increased total costs ($8913) during the treatment period, increased adverse event rates (a 1–percentage point increase in the surgery rate was associated with a 0.19–percentage point increase in the 1-year adverse event rate; β = 0.19; 95% CI, 0.09-0.27; *P* < .001), and increased mortality risk (a 1–percentage point increase in the surgery rate was associated with a 0.09–percentage point increase in the 1-year mortality rate; β = 0.09; 95% CI, 0.04-0.15; *P* < .01). Instrumental variable mortality results were even more striking for older patients and those with higher comorbidity burdens and greater frailty. Risk-adjusted estimates suggested that surgical patients had higher costs (increase of $17 278) and more adverse events (a 1–percentage point increase in the surgery rate was associated with a 0.12–percentage point increase in the 1-year adverse event rate; β = 0.12; 95% CI, 0.11 to 0.13; *P* < .001) but lower risk of mortality after PHF (a 1–percentage point increase in the surgery rate was associated with a 0.01–percentage point decrease in the 1-year mortality rate; β = −0.01; 95% CI, −0.015 to −0.005; *P* < .001).

**Conclusions and Relevance:**

This study found that higher rates of surgery for treatment of patients with PHF were associated with increased costs, adverse event rates, and risk of mortality. Orthopedic surgeons should be aware of the harms of extending the use of surgery to more clinically vulnerable patient subgroups.

## Key Points

**Question:**

Are higher rates of surgery for proximal humerus fracture associated with adverse events, mortality, and cost?

**Findings:**

In this comparative effectiveness research study of 72 823 fee-for-service Medicare beneficiaries with a proximal humerus fracture in 2011, instrumental variable analysis showed that higher rates of surgery were significantly associated with increased costs, adverse event rates, and mortality risk at 1 year. These associations were even more striking for older patients, those with higher comorbidity burdens, and those with increased frailty.

**Meaning:**

Orthopedic surgeons should be aware of the harms of extending the use of surgery to more clinically vulnerable patient subgroups.

## Teaser

This comparative effectiveness research study assesses the outcomes associated with higher rates of surgery for US Medicare beneficiaries with proximal humerus fracture, including adverse events, mortality, and costs.

## Introduction

A meta-analysis^1^ of existing randomized clinical trials suggests that the benefits and risks of surgery compared with conservative management vary, or are heterogeneous, across patients with proximal humerus fracture (PHF). The continued use of surgery over time suggests that surgeons have observed many patients improve after surgical treatment.^2,3^ In addition, lower surgery rates for more elderly patients with PHF suggest that surgeons recognize that the risks of surgery (eg, surgical complications, infections, and mortality) increase with patient complexity.^4^

If the outcomes of surgery are heterogeneous across patients, the relevant research question is not whether surgery or conservative management is the single most effective treatment for patients with PHF, but rather, what is the effective surgery rate across patients with PHF?^5,6,7^ The effective surgery rate in a population of patients with PHF is defined as the rate that results when all patients receive the treatment that is best for them.^8,9,10,11,12,13^ Can additional patients benefit from higher surgery rates, or would higher surgery rates put additional patients with PHF at unnecessary risk with little benefit? Substantial and persistent geographic variation in surgery rates for patients with PHF suggests that there is no consensus as to what the effective rate of surgery is.^2,3^

Estimates of the outcomes of surgery compared with conservative management for patients with PHF on the extensive margin are needed to address this question.^10,11,12,13,14^ Patients with PHF on the extensive margin are those who would be next to undergo surgery if surgery rates increased, or the first not to undergo surgery if surgery rates decreased. The objective of this study was to assess the outcome implications associated with higher surgery rates for Medicare patients with PHF. Instrumental variable estimators using local area surgery rates as instruments provide a natural empirical approach to address this question. Instrumental variable estimators yield estimates that are directly generalizable to patients with PHF on the extensive margin, or the set of patients with PHF whose surgery choice is sensitive to local area surgery practices.^13,15,16,17,18,19,20^ Instrumental variable estimators have been used to assess mortality after treatment for hip fracture.^21^ They are contrasted with risk-adjusted regression (RAR) estimates, which provide information on surgery outcomes for the patients who underwent surgery in our data.^13,15,16,17,18,19,20^

## Methods

### Data and Sample

This was a retrospective comparative effectiveness research cohort study of all 130 959 Medicare beneficiaries in the United States with a PHF in 2011. This project was approved by the University of South Carolina institutional review board. A waiver of informed consent was granted because this study used deidentified Medicare data. This study follows the Strengthening the Reporting of Observational Studies in Epidemiology (STROBE) reporting guideline.

Individual patients with a radiography-confirmed diagnosis of PHF in 2011 were identified using Medicare Part B carrier, outpatient, and Medpar Part A inpatient claims. eTable 1 in the Supplement contains a full list of diagnosis codes used to identify patients with PHF. The index date of PHF was defined for each beneficiary as the first date of PHF in 2011. Full cohort inclusion criteria are described in more detail in our previous work^3^ and in eTable 2 in the Supplement. Patients were required to survive the treatment period, which was the 60-day period following the index date of PHF. The full cohort of patients with PHF was stratified into subgroups to examine the differential outcomes of surgery on each subgroup of patients: aged less than 80 years vs 80 years or older, Charlson Comorbidity Index^22^ score less than 2 vs 2 or higher, and Function-Related Indicators frailty score^23^ less than 2 vs 2 or higher.

### Treatment Measures

Patients undergoing 1 of 4 surgical procedures (reverse shoulder arthroplasty, hemiarthroplasty, open reduction internal fixation, and closed reduction internal fixation) during the 60 days following the index PHF diagnosis date were classified as surgically managed patients. Surgery claims were identified using Part B carrier, outpatient, and Medpar inpatient claims files. Patients not undergoing surgery during the 60-day treatment window were classified as conservatively managed patients. Complete definitions of treatment variables are provided in eTable 3 in the Supplement.

### Outcome Measures

The outcome period began on day 61 and ran through day 365 after the index fracture. Study outcomes included the occurrence of an adverse event, mortality, and shoulder-related, non–shoulder-related, and total health care costs from Medicare’s perspective. In the year following the index fracture, any claim with at least 1 diagnosis of a surgical or medical complication was identified and defined as an adverse event. Adverse events were defined as medical and surgical complications typically associated with elderly adults or surgical repair of PHF and included infection, nerve injury, prosthetic complication, hematoma, avascular necrosis, adhesive capsulitis, shoulder instability or dislocation, pneumonia, cardiac dysrhythmias, congestive heart failure, and deep vein thrombosis or pulmonary embolism.^4^ Mortality dates were obtained from the 2011 and 2012 Medicare Beneficiary summary files. The mortality variable was calculated during the 305-day period following the 60-day treatment window, which equaled 1 if the patient died during the period, and equaled 0 otherwise.

Health care costs were summed across all Part A and Part B claims during 2 distinct outcome periods. The treatment window represented the 60-day period beginning on the day of the index fracture and ending 60 days thereafter (days 0-60). The outcome period began on day 61 and ran through day 365. Those claims with a diagnosis of 1 of 192 shoulder diagnoses were deemed a shoulder-related health care cost. All other claims without a shoulder diagnosis were categorized as non–shoulder-related costs. Total costs were a sum of shoulder-related and non–shoulder-related costs. All costs were from the perspective of the Medicare program and included payment amounts made by Medicare to health care professionals.

### Covariates

The list of all patient demographic and clinical covariates specified in all estimation equations can be found in eTable 4 in the Supplement and in a previous publication.^3^ Demographic characteristics included age, sex, race, dual eligibility, Charlson Comorbidity Index score, and Frailty Risk Index score. Clinical covariates included previous-year shoulder diagnoses of osteoarthritis, rheumatoid arthritis, rotator cuff arthropathy, and avascular necrosis, as well as Medicare spending in the previous year.

### Analytical Approach

It is well known that different estimation approaches for observational data yield average treatment effect estimates over distinct subgroups of patients and that these approaches rely on different assumption sets to avoid confounding bias.^10,15,17,18,20,24,25,26,27,28,29,30,31^ Consequently, when treatment outcomes are heterogeneous across patients, estimates from these approaches can differ and reveal different information about the study population.^20,31^ Here we used instrumental variable estimators to assess our research question. The RAR estimators were provided for comparison. Table 1 summarizes the properties of these estimators when assessing the outcomes of surgery compared with conservative management. Instrumental variable estimators yield the average surgery effect for the subgroup of the patients whose surgery choices were sensitive to the instrumental variable used in the analysis, which is known as the local average treatment effect in the instrumental variable literature.^24,26^ To be unbiased, instrumental variable estimators assume that the distribution of potential outcomes under conservative management are the same for patients with different instrumental variable values.^24,26^ The RAR estimators yield estimates of the average surgery effect for the subgroup of the patients who chose surgery (eg, the average treatment effect on the treated).^10,17,30,32^ For the estimate of average treatment effect on the treated to be unbiased, RAR estimators assume that the distribution of potential outcomes under conservative management are the same for patients ultimately treated with either surgery or conservative management.^24,26^

**Table 1.** Assumptions Required and Treatment Effect Parameters Estimated for Risk-Adjusted Regression and Instrumental Variable Estimators

Estimator	Treatment Effect Parameter Estimated	Assumptions Required to Eliminate Confounding Bias
Risk-adjusted regression	Average effect of surgery compared with conservative management for the subgroup of patients who underwent surgery (average treatment effect on the treated)	Distribution of potential outcomes under conservative management are the same for patients ultimately treated with either surgery or conservative management
Instrumental variables	Average effect of surgery compared with conservative management for the subgroup of patients whose surgery choices were sensitive to the instrumental variable specified (local average treatment effect)	Distribution of potential outcomes under conservative management are the same for patients with different instrumental variable values

The instrumental variables for our instrumental variable models were based on measures of local area practice styles that have been a practical and rich source for instrument development.^10,11,14,33,34,35,36,37,38,39,40,41,42,43,44^ The use of local area practice style measures as an instrument enabled a direct estimate of the outcomes of higher surgery rates on study outcomes. We used quintiles of risk-adjusted area surgery ratios (ASRs) across hospital referral regions (HRRs) as measures of local area practice styles. The ASRs were calculated for each HRR as the ratio of the number of patients with PHF in an HRR who underwent surgical treatment over the sum across these patients of their estimated probabilities of undergoing surgery. Estimated probabilities for each patient were produced from a logistic regression model of surgery choice as a function of measured covariates over the entire sample. Measured patient covariates in the model of surgery choice can be found in eTable 4 in the Supplement. The range of ASRs across all HRRs was divided into 5 quintiles, which became our instruments. More details on the construction of our instruments can be found in the eAppendix in the Supplement. Surgeons in HRRs with larger ASR values are theorized to have stronger beliefs as to the effectiveness of surgery compared with conservative management for patients with PHF. Patients in our sample were assigned the surgery rate and ASR value according to their residence HRR. Instrumental variable analysis was conducted using the procedure IVREG2 in Stata statistical software version 13.1 (StataCorp). All models controlled for patient demographic and clinical characteristics. We used RAR models to estimate the association between surgery and each study outcome after controlling for measured patient covariates associated with comorbidities and frailty. The RAR analysis was conducted using the Stata procedure REG.

### Statistical Analysis

Descriptive statistics summarizing patient characteristics for surgery and conservative management groups were assessed by the 2-sample independent *t* test for continuous variables and Pearson χ^2^ test for categorical data. The Cochrane-Armitage test was used to assess trends of patients grouped by ASR quintiles.^45,46^ A 2-sided *P* < .05 was considered statistically significant. *F* statistics testing whether instruments described a significant amount of variation in surgery rates were assessed for the sample population and subgroups according to age, comorbidities, and frailty. An *F* statistic greater than 10 is considered indicative of a strong instrument.^47^ SAS statistical software version 9.4 (SAS Institute) was used for building our analytic database, and Stata was used for statistical analyses. Sensitivity analyses were conducted to assess the impact of cohort inclusion criteria and data extremes on the robustness of our findings and study conclusions. Data analysis was performed January through June 2019.

## Results

### Sample Characteristics

The final cohort included 72 823 patients (mean [SD] age, 80.0 [7.9] years; 13 958 [19.2%] men). In the subgroups, 36 216 patients were younger than 80 years, 36 607 patients were aged 80 years or older, 33 389 patients had a Charlson Comorbidity Index score less than 2, 39 434 patients had a Charlson Comorbidity Index score of 2 or higher, 44 820 patients had a Function-Related Indicators score less than 2, and 28 003 patients had a Function-Related Indicators score of 2 or higher.

Surgery within 60 days of diagnosis was used for 16.3% of the study cohort, and when compared with conservatively managed patients, surgically treated patients were younger (mean [SD] age, 80.4 [8.1] years vs 78.0 [7.2] years; *P* < .001), had fewer comorbidities (Charlson Comorbidity Index score of 0, 14 863 [24.4%] patients vs 3468 [29.1%] patients; *P* < .001), and had a lower Function-Related Indicators score (Function-Related Indicator score of 0, 20 720 [34.0%] patients vs 4980 [41.8%] patients; *P* < .001). Women (9835 [82.5%] patients vs 49 030 [80.5%] patients; *P* < .001) and white beneficiaries (11 388 [95.1%] patients vs 56 663 [93.0%] patients; *P* < .001) were more likely to be treated surgically than conservatively managed, and a lower percentage of surgical patients were dual eligible for Medicaid the month of their index fracture compared with conservatively managed patients (1086 [9.1%] patients vs 8728 [14.3%] patients; *P* < .001). A lower percentage of surgical patients, compared with conservatively managed patients, had a history of shoulder conditions (osteoarthritis, 2819 [23.6%] patients vs 15 556 [25.5%] patients [*P* < .001]; rheumatoid arthritis, 886 [7.4%] vs 4890 [8.0%] patients [*P* = .02]; rotator cuff arthropathy, 752 [6.3%] vs 4077 [6.7%] patients [*P* = .06]). Surgical patients had lower Medicare cost in the year preceding the index fracture compared with conservatively managed patients (mean [SD], $11 833 [$20 403] vs $15 472 [$26 365]; *P* < .001). Table 2 also shows average unadjusted 1-year outcomes for the full cohort by treatment group. Surgically managed patients experienced more adverse events (6979 [58.5%] patients vs 30 934 [50.8%] patients; *P* < .001) and had higher shoulder-related (mean [SD], $15 065 [$10 745] vs $3306 [$6330]; *P* < .001) and non–shoulder-related (mean [SD], $27 584 [$32 563] vs $23 690 [$32 308]; *P* < .001) health care costs in the year following their index diagnosis compared with conservatively managed patients. A larger proportion of conservatively managed patients died in the outcome period compared with surgically managed patients (10.8% vs 7.0%; *P* < .001).

**Table 2.** Medicare Proximal Humerus Fracture Patient Characteristics by Treatment Group

Characteristic	Patients, No. (%)			*P* Value^a^
	Total Population (N = 72 823)	Surgical Management (n = 11 922)	Conservative Management (n = 60 901)	
Male	13 958 (19.2)	2087 (17.5)	11 871 (19.5)	<.001
Age, mean (SD), y	80.0 (7.9)	78.0 (7.2)	80.4 (8.1)	<.001
Age group, y				
66-69	9792 (13.4)	2039 (17.1)	7753 (12.7)	<.001
70-75	15 344 (21.1)	3080 (25.8)	12 264 (2.1)	
76-79	11 080 (15.2)	2120 (17.8)	8960 (14.7)	
80-85	18 107 (24.9)	2831 (23.7)	15 276 (25.1)	
≥86	18 500 (25.4)	1852 (15.5)	16 684 (27.3)	
Race/ethnicity				
Asian	667 (0.9)	82 (0.7)	585 (1.0)	<.001
Black	2270 (3.1)	243 (2.0)	2027 (3.3)	
Hispanic	989 (1.4)	125 (1.0)	864 (1.4)	
Other	896 (1.2)	134 (1.1)	762 (1.3)	
White	68 001 (93.4)	11 388 (95.1)	56 663 (93.0)	
Fully dual eligible^b^	9814 (13.5)	1086 (9.1)	8728 (14.3)	<.001
Charlson Comorbidity Index score^22^				
0	18 331 (25.2)	3468 (29.1)	14 863 (24.4)	<.001
1	15 058 (20.7)	2586 (21.7)	12 472 (20.5)	
2	11 373 (15.6)	1787 (15.0)	9586 (15.7)	
3	8984 (12.3)	1454 (12.2)	7530 (12.4)	
≥4	19 077 (26.2)	2627 (22.0)	16 540 (27.0)	
Function-Related Indicator score^23^				
0	25 682 (35.3)	4980 (41.8)	20 720 (34.0)	<.001
1	19 138 (26.3)	3231 (27.1)	15 907 (26.1)	
2	11 445 (15.7)	1729 (14.5)	9716 (16.0)	
≥3	16 558 (22.7)	1982 (16.6)	14 576 (23.9)	
Previous-year shoulder diagnoses				<.001
Osteoarthritis	18 375 (25.2)	2819 (23.6)	15 556 (25.5)	<.001
Rheumatoid arthritis	5776 (7.9)	886 (7.4)	4890 (8.0)	.02
Rotator cuff arthropathy	4829 (6.6)	752 (6.3)	4077 (6.7)	.06
Avascular necrosis	120 (0.2)	30 (0.3)	90 (0.1)	.03
Previous-year Medicare cost, mean (SD), $^c^	14 877 (25 520)	11 833 (20 403)	15 472 (26 365)	<.001
Outcomes at 1 y				
Adverse event^d^	37 913 (52.1)	6979 (58.5)	30 934 (5.8)	<.001
Costs, mean (SD), $				
Total^e^	29 559 (34 273)	42 649 (35 353)	26 998 (33 464)	<.001
Shoulder related^f^	5231 (8446)	15 065 (10 745)	3306 (6330)	<.001
Non–shoulder related^g^	24 327 (32 382)	27 584 (32 563)	23 690 (32 308)	<.001
Mortality in 61- to 365-d period	7431 (10.2)	839 (7.0)	6592 (10.8)	<.001

^a^ Differences across groups were assessed by the 2-sample independent *t* test for continuous variables and Pearson χ^2^ test for categorical data.

^b^ Beneficiary was fully dual-eligible for Medicare and Medicaid during the month of the index fracture.

^c^ Total Part A and B payments made by Medicare for the beneficiary over the period of 365 days before their index fracture date.

^d^ Adverse events include pneumonia, cardiac dysrhythmias, congestive heart failure, deep vein thrombosis or pulmonary embolism, infection, nerve injury, prosthetic complication, hematoma, avascular necrosis, adhesive capsulitis, and instability or dislocation.

^e^ Total Part A and B payments made by Medicare for the beneficiary over the 365-day period following index proximal humerus fracture date.

^f^ Total Part A and B shoulder-related payments made by Medicare for the beneficiary over the 365-day period following index proximal humerus fracture date.

^g^ Total Part A and B non–shoulder-related payments made by Medicare for the beneficiary over the 365-day period following index proximal humerus fracture date.

The proportion of patients treated surgically ranged from 1.8% to 33.3% across HRRs in the United States. Table 3 compares patient characteristics across ASR quintiles, which served as our instrumental variable. The mean percentage of patients who underwent surgery after PHF varied from 10.6% to 22.5% from the lowest to highest ASR quintile. Areas with higher surgery rates had higher rates of adverse events (53.6% vs 52.1%; *P* < .001) and higher mortality rates (10.5% vs 9.8%; *P* = .16) compared with areas with the lowest surgery rates. Measured patient factors associated with study outcomes, such as Charlson Comorbidity Index score, Function-Related Indicators score, and age, were similar across ASR quintiles. Although some trends in patient characteristics across ASR quintiles did reach statistical significance (quintile 1 vs quintile 5, 2.6% vs 3.3% for black race [*P* < .01]; 1.0% vs 0.9% for Hispanic ethnicity [*P* = .02]; 14.9% vs 11.0% for dual eligibility [*P* < .001]; and 23.9% vs 26.4% for osteoarthritis [*P* < .001]), the absolute differences across quintiles were small in comparison with the differences in the surgically and conservatively managed patients in Table 2.

**Table 3.** Characteristics of Medicare Patients With Proximal Humerus Fracture in 2011 by Local Area Surgery Ratios

Characteristic	Patients, % (N = 72 823)	Quintile of Surgical Management Area Surgery Ratios, Patients, %					*P* Value^a^
		1 (n = 14 909)	2 (n = 14 227)	3 (n = 14 591)	4 (n = 15 450)	5 (n = 13 646)	
Area surgery ratio, mean	1	0.66	0.88	0.98	1.13	1.35	
Surgery %, mean	16.3	10.6	14.4	16.1	18.5	22.5	
Surgery % range across hospital referral regions	1.8-33.3	1.9-14.2	12.5-16.3	14.5-18.0	16.4-20.9	18.5-33.3	
Male	19.2	19.7	19.3	19.0	18.9	19.0	.06
Age, mean, y	80.0	80.4	79.9	79.9	79.9	79.8	<.001
Age group, y							
66-69	13.4	12.4	13.9	13.6	13.8	13.4	.10
70-75	21.1	19.8	21.0	21.4	21.1	22.1	<.001
76-79	15.2	15.3	15.2	15.1	15.2	15.4	.61
80-85	24.9	25.6	24.2	25.2	24.6	24.7	.16
≥86	25.4	26.8	25.7	24.7	25.3	24.4	<.001
Race/ethnicity							
Asian	0.9	0.8	0.6	1.2	1.5	0.3	.87
Black	3.1	2.6	3.4	2.9	3.4	3.3	<.001
Hispanic	1.4	1.0	2.1	1.0	1.8	0.9	.02
Other	1.2	1.1	1.3	1.5	1.5	0.8	.07
White	93.4	94.5	92.6	93.4	91.8	94.7	.90
Fully dual eligible^b^	13.5	14.9	13.3	13.2	14.8	11.0	<.001
Charlson Comorbidity Index score^22^							
0	25.2	24.8	25.8	24.8	26.0	24.4	.62
1	20.7	20.6	20.6	21.0	20.9	20.3	.67
2	15.6	15.3	15.7	16.2	15.1	15.9	.79
3	12.3	12.5	12.2	12.2	12.0	12.8	.79
≥4	26.2	26.8	25.7	25.8	26.0	26.7	.75
Function-Related Indicator score^23^							
0	35.3	34.9	36.1	35.3	35.3	34.4	.37
1	26.3	26.7	26.1	26.2	25.7	26.7	.76
2	15.7	16.0	15.5	15.2	15.8	16.2	.88
≥3	22.7	22.4	22.3	23.3	23.0	22.6	.23
Previous-year shoulder diagnoses							
Osteoarthritis	25.2	23.9	24.2	25.8	25.9	26.4	<.001
Rheumatoid arthritis	7.9	8.4	7.9	8.0	7.6	7.8	.05
Rotator cuff arthropathy	6.6	6.7	6.5	6.2	6.5	7.2	.02
Avascular necrosis	0.2	0.2	0.2	0.2	0.2	0.2	.79
Days to surgery	7.9	8.5	8.1	8.2	7.3	7.7	<.001
Surgical procedure							<.001
Hemiarthroplasty	22.2	25.3	24.9	22.7	21.0	19.6	
Open reduction internal fixation	65.9	64.5	64.9	65.7	67.8	65.6	
Reverse shoulder arthroplasty	11.4	9.5	10.1	10.9	10.7	14.3	
Previous-year Medicare cost, mean, $^c^	14 877	15 564	14 607	14 731	14 776	14 679	<.001
Outcomes at 1 y							
Adverse event^d^	52.1	52.1	51.1	51.5	52.0	53.6	<.001
Costs, mean, $							
Total^e^	29 559	30 185	28 659	29 002	29 615	30 342	<.001
Shoulder related^f^	5231	5177	4996	5009	5383	5598	<.001
Non–shoulder related^g^	24 327	25 007	23 662	23 992	24 231	24 743	<.001
Mortality in 61- to 365-d period	10.2	9.8	10.4	10.0	10.4	10.5	.16

^a^ Cochrane-Armitage test was used to assess trends across area surgery ratio quintiles.

^b^ Beneficiary was fully dual-eligible for Medicare and Medicaid during the month of the index fracture.

^c^ Total Part A and B payments made by Medicare for the beneficiary for the 365 days before the index fracture date.

^d^ Adverse events include pneumonia, cardiac dysrhythmias, congestive heart failure, deep vein thrombosis or pulmonary embolism, infection, nerve injury, prosthetic complication, hematoma, avascular necrosis, adhesive capsulitis, and instability or dislocation.

^e^ Total Part A and B payments made by Medicare for the beneficiary for the 365-day period after index proximal humerus fracture date.

^f^ Total Part A and B shoulder-related payments made by Medicare for the beneficiary for the 365-day period after index proximal humerus fracture date.

^g^ Total Part A and B non–shoulder-related payments made by Medicare for the beneficiary for the 365-day period after index proximal humerus fracture date.

### Instrumental Variable Results

Table 4 shows instrumental variable estimates for the clinical outcomes. Table 4 also shows the *F* statistic assessing the effects of our instrumental variable on surgery choice. Instruments with an *F* statistic less than 10 are considered weak and more susceptible to bias in the instrumental variable literature.^48^ All *F* statistics in this study are much greater than 10 (total cohort, 205.9; age <80 years, 98.9; age ≥80 years, 111.3; Charlson Comorbidity Index score <2, 95.2; Charlson Comorbidity Index score ≥2, 110.9; Function-Related Indicators score <2, 136.3; Function-Related Indicators score ≥2, 69.9), signifying that local area treatment signatures, measured by risk-adjusted ASRs, were strong instruments. Table 4 shows the mean percentage of patients undergoing surgery in each patient subgroup and the corresponding range in rates across the ASRs. Our instrumental variable surgery effect estimates directly stem from the variation in surgery rates within these ranges. In addition, the surgery rates in Table 4 for stratified subsets of our sample show that patients with PHF who were older, had higher comorbidity burdens, and greater frailty were less likely to undergo surgery (mean [range], age <80 years, 19.9% [13.9%-26.4%] vs age ≥80 years, 12.8% [7.7%-18.4%]; Charlson Comorbidity Index score <2, 18.1% [12.2%-24.4%] vs Charlson Comorbidity Index score ≥2, 14.9% [9.4%-20.9%]; Function-Related Indicators score <2, 18.3% [12.1%-24.9%] vs Function-Related Indicators score ≥2, 13.2% [8.3%-18.6%]).

**Table 4.** Risk-Adjusted Regression and Instrumental Variable Estimates of the Association of Surgery With Adverse Events and Mortality for Medicare Patients With Proximal Humerus Fracture in 2011 and Stratified Subgroups

Estimate	*F* Statistic	Surgery Rate Across Hospital Referral Regions, Mean (Range), %	Adverse Event Rate, %^a^	Mortality Rate for 0-365 d, %
Instrumental variable estimates				
Total cohort (N = 72 823)	205.9^b^	16.3 (10.6-22.5)	0.19^b^	0.09^c^
Age <80 y (n = 36 216)	98.9^b^	19.9 (13.9-26.4)	0.16^c^	0.10^d^
Age ≥80 y (n = 36 607)	111.3^b^	12.8 (7.7-18.4)	0.20^c^	0.11^b^
Charlson Comorbidity Index score^22^				
<2 (n = 33 389)	95.2^b^	18.1 (12.2-24.4)	0.13^e^	0.03
≥2 (n = 39 434)	110.9^b^	14.9 (9.4-20.9)	0.24^b^	0.15^c^
Function-Related Indicators score^23^				
<2 (n = 44 820)	136.3^b^	18.3 (12.1-24.9)	0.16^c^	0.09^c^
≥2 (n = 28 003)	69.9^b^	13.2 (8.3-18.6)	0.22^c^	0.11^e^
Risk-adjusted estimates				
Total cohort (N = 72 823)		16.3 (10.6-22.5)	0.12^b^	−0.01^b^
Age <80 y (n = 36 216)		19.9 (13.9-26.4)	0.13^b^	−0.01^c^
Age ≥80 y (n = 36 607)		12.8 (7.7-18.4)	0.11^b^	−0.02^b^
Charlson Comorbidity Index score^22^				
<2 (n = 33 389)		18.1 (12.2-24.4)	0.14^b^	−0.01^b^
≥2 (n = 39 434)		14.9 (9.4-20.9)	0.11^b^	−0.01^e^
Function-Related Indicators score^23^				
<2 (n = 44 820)		18.3 (12.1-24.9)	0.13^b^	−0.00^e^
≥2 (n = 28 003)		13.2 (8.3-18.6)	0.11^b^	−0.02^b^

^a^ Adverse event includes pneumonia, cardiac dysrhythmias, congestive heart failure, deep vein thrombosis or pulmonary embolism, infection, nerve injury, prosthetic complication, hematoma, avascular necrosis, adhesive capsulitis, and instability or dislocation.

^b^ *P* < .001.

^c^ *P* < .01.

^d^ *P* < .05.

^e^ *P* < .10.

Instrumental variable estimates show that higher surgery rates for PHF were associated with higher adverse event rates and higher mortality in the full cohort and select patient subsets. These instrumental variable estimates show the absolute average effect of a 1–percentage point increase in surgery rates on study outcome rates. For example, the instrumental variable estimate for 1-year adverse event risk for the full cohort implies that a 1–percentage point increase in the surgery rate was associated with a 0.19–percentage point increase in the 1-year adverse event rates (β = 0.19; 95% CI, 0.09-0.27; *P* < .001). The instrumental variable estimate for 1-year mortality for the full cohort implies that a 1–percentage point increase in the surgery rate was associated with a 0.09–percentage point increase in the 1-year mortality rates (β = 0.09; 95% CI, 0.04-0.15; *P* < .01). Furthermore, the adverse event and mortality risks associated with higher surgery rates were greatest in older patients and those with higher comorbidity burdens and frailty index scores. For example, the instrumental variable estimate for 1-year adverse event risk for the patients with higher comorbidity burdens (Charlson Comorbidity Index score ≥2) suggests that a 1–percentage point increase in surgery rates was associated with a 0.24–percentage point increase in the 1-year adverse event rates (β = 0.24; 95% CI, 0.11-0.36; *P* < .001) and a 0.15–percentage point increase in 1-year mortality rates (β = 0.15; 95% CI, 0.06-0.24; *P* < .01). Among the subset of patients with a Function-Related Indicator score of 2 or higher, a 1–percentage point increase in the surgery rate was associated with a 0.22–percentage point increase in the 1-year adverse event rate and a 0.11–percentage point increase in the 1-year mortality rate. Among the subset of patients aged 80 years or older, a 1–percentage point increase in the surgery rate was associated with a 0.20–percentage point increase in the 1-year adverse event rate and a 0.11–percentage point increase in the 1-year mortality rate.

Table 5 shows instrumental variable estimates for cost outcomes. During the 60-day treatment period, shoulder-related (β = $5358), non–shoulder-related (β = $3555), and total (β = $8913) costs were higher in local areas with higher surgery rates in the full cohort and most subgroups. During the outcome period, higher surgery rates were associated with lower shoulder-related, non–shoulder-related, and total costs for the full cohort and most patient subgroups, although most estimates did not reach significance. These results were especially pronounced in the more clinically vulnerable patient subgroups, which showed the greatest reductions in cost, although the estimates did not reach statistical significance.

**Table 5.** Risk-Adjusted Regression and Instrumental Variable Estimates for the Association Between Surgery Utilization and Cost Outcomes for Medicare Patients With Proximal Humerus Fracture in 2011 and Stratified Subgroups

Stratified Models	Cost Outcomes, $					
	Treatment Period^a^			Outcome Period^b^		
	Shoulder^c^	Non-Shoulder^d^	Total^e^	Shoulder	Non-Shoulder	Total
Instrumental variables estimates						
Total cohort (N = 72 823)	5358^f^	3555^g^	8913^f^	−983^f^	−2179	−3117
Age <80 y (n = 36 216)	5906^f^	4598^g^	10 505^f^	−1583^f^	−3113	−4696
Age ≥80 y (n = 36 607)	4590^f^	1755	6345^g^	−157	−1734	−1891
Charlson Comorbidity Index score^22^						
<2 (n = 33 389)	4722^f^	414	5137^g^	−681^g^	−1792	−2473
≥2 (n = 39 434)	5940^f^	6352^h^	12 292^f^	−1166^h^	−2810	−3976
Function-Related Indicators score^23^						
<2 (n = 44 820)	5678^f^	2970^i^	8648^f^	−1047^f^	255	−798
≥2 (n = 28 003)	4543^f^	4278	8822^g^	−700	−8905^i^	−9605^i^
Risk-adjusted estimates						
Total cohort (N = 72 823)	11 378^f^	5900^f^	17 278^f^	621^f^	919^f^	1541^f^
Age <80 y (n = 36 216)	10 528^f^	5211^f^	15 740^f^	678^f^	955^f^	1633^f^
Age ≥80 y (n = 36 607)	12 498^f^	6708^f^	19 206^f^	557^f^	1148^f^	1706^f^
Charlson Comorbidity Index score^22^						
<2 (n = 33 389)	10 702^f^	4995^f^	15 697^f^	623^f^	600^g^	1223^f^
≥2 (n = 39 434)	12 048^f^	6798^f^	18 847^f^	620^f^	1269^f^	1889^f^
Function-Related Indicators score^23^						
<2 (n = 44 820)	11 030^f^	5342^f^	16 373^f^	613^f^	885^f^	1498^f^
≥2 (n = 28 003)	12 103^f^	7020^f^	19 123^f^	641^f^	990^g^	1632^f^

^a^ Treatment period is days 0 to 60.

^b^ Outcome period is days 61 to 365.

^c^ Part A and B shoulder-related payments made by Medicare for the beneficiary.

^d^ Part A and B non–shoulder-related payments made by Medicare for the beneficiary.

^e^ Total Part A and B shoulder-related and non–shoulder-related payments made by Medicare for the beneficiary.

^f^ *P* < .001.

^g^ *P* < .05.

^h^ *P* < .01.

^i^ *P* < .10.

### Risk-Adjusted Results

Table 4 shows RAR estimates on clinical outcomes for the full sample and patient subgroups. In the full cohort and across all subgroups of patients with PHF, the probability of adverse events was higher for the surgically managed patients (a 1–percentage point increase in the surgery rate was associated with a 0.12–percentage point increase in the 1-year adverse event rate; β = 0.12; 95% CI, 0.11 to 0.13; *P* < .001). Across all subgroups, the risk-adjusted estimates suggest that surgery was associated with a decrease in the 1-year mortality rate compared with conservative management for the patients who underwent surgery. In the full cohort, a 1–percentage point increase in the surgery rate was associated with a 0.01–percentage point decrease in the 1-year mortality rate (β = −0.01; 95% CI, −0.015 to −0.005; *P* < .001).

In Table 5, shoulder-related (β = $621), non–shoulder-related (β = $919), and total (β = $1541) costs were all higher among patients undergoing surgery compared with conservatively managed patients across all periods and subgroups. In the treatment period, surgical patients had total Medicare reimbursements that were a mean of $17 278 higher than those for conservatively managed patients, the majority of which was likely due to the surgical procedure. Shoulder-related, non–shoulder-related, and total costs were also higher for surgery recipients in the outcome period. Sensitivity analyses were conducted to assess the impact of cohort inclusion criteria and data extremes on the robustness of our findings but did not yield materially different conclusions.

## Discussion

Our analysis showed that higher rates of surgery were associated with increased 1-year mortality and adverse event rates. This finding suggests that surgery rates in 2011 for patients with PHF were higher than the effective rate and that a reduction in surgery rates could reduce mortality and adverse event rates for patients with PHF. This result is especially pronounced for older cohorts and those with higher comorbidity burdens and increased frailty. Similar to more well-studied fracture populations, such as patients with hip fracture,^49,50^ patients with PHF are an especially frail population, and the surgery decision is associated with a series of events that can trigger poor outcomes, including death. Our instrumental variable estimates are unbiased if unmeasured patient differences between surgically and conservatively managed patients are unrelated to the instrument we selected. Consistent distributions in measured patient characteristics across patients grouped by our instrument in Table 3 support this assumption.

For comparison, we used risk-adjusted estimators to estimate the association between surgery and each study outcome by controlling for a series of measured baseline covariates. Risk-adjusted estimators provide estimates of the outcomes of surgery for the patients who undergo surgery. For risk-adjusted estimates to be unbiased, outcomes for surgical patients if they had not undergone surgery would have to be the same as those observed for the conservatively treated patients.^24,26^ Risk-adjusted estimates suggested that surgical patients had lower risks of mortality after PHF. However, we found meaningful differences in patient age, comorbidity burden, and frailty index between surgically and conservatively managed patients, which suggests that there are likely additional unmeasured differences between treatment groups that are partly responsible for the estimated treatment outcomes we observed. Given that patients with PHF undergoing surgery are younger, less frail, and have lower comorbidity burdens, it is likely that the estimates of reduced mortality from surgery are biased high as the result of unmeasured differences between the patients selected for surgery and conservative care.

### Limitations

Our results should be viewed through the limitations of the study design. First, our adverse event and mortality instrumental variable estimates should be interpreted only within the surgery rate ranges we provided in Table 4. Because surgery outcomes are likely heterogeneous across patients with PHF, it is risky to generalize our estimates to surgery rates below or above these surgery rate ranges. Our study sample was from 2011, and surgery rates for PHF continue to change. The survival implications associated with surgery rate changes from different baseline rates should be evaluated. Second, the *International Classification of Diseases, Ninth Revision* diagnosis codes available for this sample of Medicare data likely does not include information on many factors associated with patient survival, which are also associated with the choice of surgery, such as fracture complexity. Because, patients with complex fractures are more likely to undergo surgery, a portion of the mortality associated with surgery in our instrumental variable models will stem from differences in the distributions of fracture complexity across local areas. However, previous research^38^ suggests that this bias risk is attenuated when large geographic areas such as the HRRs are used, as we did here. However, future research using Medicare claims after 2015, which use *International Statistical Classification of Diseases and Related Health Problems, Tenth Revision* diagnosis codes that reflect fracture complexity, is needed to validate this assumption. Third, and related to our second limitation, is the assumption that patients are similar across quintiles of our instrument. Our instrumental variable estimates are unbiased if unmeasured patient differences between surgically and conservatively managed patients are not associated with the instrument we selected. Although some minor differences in shoulder health were noted across instrument quintiles, mostly consistent distributions in measured patient characteristics across patients grouped by our instrument in Table 3 support our assumption that patients were similar across high and low surgery areas. However, it must still be acknowledged that patient differences across areas, if they exist, could confound our findings. Fourth, we chose to exclude patients who did not survive the treatment period. Therefore, our results do not generalize to those patients who were very frail and not survive the 60 days following their fracture.

## Conclusions

This study included a diverse, national sample of patients with PHF. We found wide treatment variation and sought to understand the implications associated with higher rates of surgery for patients with PHF on patient outcomes. Instrumental variable methods offer an approach to directly assess this question using populations of Medicare patients with PHF from across local areas.^13,15,16,17,18,19,20^ Our estimates provide evidence to suggest that current PHF surgery rates are higher than the effective surgery rate and that lowering surgery rates may be associated with lower 1-year mortality rates and adverse event risks. These results are especially pronounced for patients with PHF in high-risk patient subgroups. Because the choice of treatment can affect a number of measured and unmeasured variables, some of which could not be measured in this analysis, further analysis using patient-reported outcomes is warranted.

## Notes

Supplement. **eTable 1.** Proximal Humerus Fracture Diagnosis Codes **eTable 2.** Medicare 2011 Proximal Humerus Fracture Sample Inclusion Criteria **eTable 3.** Treatment Groups **eTable 4.** Analysis Covariate List and Definitions **eAppendix.** Instrument Development
