# Supplementary material for: Association of Surgical Treatment With Adverse Events and Mortality Among Medicare Beneficiaries With Proximal Humerus Fracture
Source: JAMA Netw Open. 2020 Jan 10;3(1):e1918663. doi: 10.1001/jamanetworkopen.2019.18663 (PMC6991245; doi:10.1001/jamanetworkopen.2019.18663)
Supplement: Supplement. — eTable 1. Proximal Humerus Fracture Diagnosis Codes eTable 2. Medicare 2011 Proximal Humerus Fracture Sample Inclusion Criteria eTable 3. Treatment Groups eTable 4. Analysis Covariate List and Definitions eAppendix. Instrument Development [file jamanetwopen-3-e1918663-s001.pdf]

Supplementary Online Content

Floyd SB, Thigpen C, Kissenberth M, Brooks JM. Association of surgical treatment with adverse events and mortality among Medicare beneficiaries with proximal humerus fracture. *JAMA Netw Open*. 2020;3(1):e1918663. doi:10.1001/jamanetworkopen.2019.18663

eTable 1. Proximal Humerus Fracture Diagnosis Codes

eTable 2. Medicare 2011 Proximal Humerus Fracture Sample Inclusion Criteria

eTable 3. Treatment Groups

eTable 4. Analysis Covariate List and Definitions

eAppendix. Instrument Development

This supplementary material has been provided by the authors to give readers additional information about their work.

© 2020 Floyd SB et al. *JAMA Network Open.*

eTable 1. Proximal Humerus Fracture Diagnosis Codes

Diagnosis groups	ICD-9-CM Diagnosis codes
Proximal Humerus Fracture	812.00, 812.01, 812.02, 812.03, 812.09, 812.10, 812.11, 812.12, 812.13, 812.19

© 2020 Floyd SB et al. *JAMA Network Open.*

eTable 2. Medicare 2011 Proximal Humerus Fracture Sample Inclusion Criteria

**Inclusion Criteria**	**N**
Medicare Part B carrier (physician services), outpatient, or medpar (inpatient) claims with a proximal humerus fracture diagnosis from January 1, 2011-December 31, 2011 (ICD-9 Diagnosis codes: 812.00, 812.01, 812.02, 812.03, 812.09, 812.10, 812.11, 812.12, 812.13, 812.19) (Index diagnosis)	130,959
No Part B carrier, outpatient, or medpar claims with proximal humerus fracture diagnosis in 365-days before the index diagnosis in 2011	107,838
Shoulder x-ray claim (HCPCS codes: 73000, 73010, 73020, 73030, 73050, 73060) in Part B carrier or outpatient revenue center claims within 7 days of index diagnosis (x-ray claim can occur before or after index diagnosis)	95,229
No Part B carrier, outpatient, or medpar claims with a diagnosis of clavicle or hip fracture within 7 days of index diagnosis	86,147
No Part B carrier, outpatient, or medpar claims with total joint replacement procedure in 365-days before the index diagnosis in 2011	85,841
Age 66+ at index diagnosis	84,589
Located within continental United States or Hawaii	84,399
Continuously enrolled in Medicare Parts A and B and never enrolled in HMO, from 365-days prior to index to 365-days after index diagnosis	77,075
Complete HRR data	77,053
Survived the treatment period (0-60 days) following index fracture	72,823

© 2020 Floyd SB et al. *JAMA Network Open.*

eTable 3. Treatment Groups

Surgery Group	ICD-9-CM Procedure codes	HCPCS Codes
Hemiarthroplasty	81.81	23470, 23616
Reverse Shoulder Arthroplasty	81.88	23472
ORIF	79.31	23615, 23630, 23670, 23680
CRIF	79.11	24515

© 2020 Floyd SB et al. *JAMA Network Open.*

eTable 4. Analysis Covariate List and Definitions

Covariate	Definition
Patient demographics	
Gender	Male, Female
Age Group	66-69, 70-75, 76-79, 80-85, 86+
Race*	Asian, Black, Hispanic, Other, White
Fully Dual Eligible	Beneficiary was fully dual-eligible for Medicare and Medicaid during the month of the index fracture
Charlson Comorbidity Index	0, 1, 2, 3, ≥4
Frailty Risk Index (FRI)	0, 1, 2, ≥3
Previous year shoulder diagnoses	
Osteoarthritis	A diagnosis on a carrier claim over the period of 365-days prior to their index fracture date (ICD-9 codes: 715.00, 715.09, 715.10, 715.12, 715.20, 715.21, 715.22, 715.30, 715.31, 715.32, 715.80, 715.89, 715.90, 715.91, 715.92).
Rheumatoid Arthritis	A diagnosis on a carrier claim over the period of 365-days prior to their index fracture date (ICD-9 codes: 274.00, 274.01, 274.02, 274.03, 274.10, 696.0, 701.0, 710.1, 711.01, 711.02, 711.09, 714.0, 714.1, 714.2, 714.30, 714.31, 714.32, 714.33, 714.4, 714.89, 714.9, 719.21, 719.22, 719.29, 719.31, 719.32, 719.39, 725, 729.0)
Rotator Cuff Arthropathy	A diagnosis on a carrier claim over the period of 365-days prior to their index fracture date (ICD-9 codes: 726.10, 726.13, 726.19, 727.60, 727.61, 728.83, 840.3, 840.4, 840.5, 905.8, 716.10, 716.11, 716.12, 716.19, 716.41, 716.42, 716.49, 716.80, 716.81, 716.82, 716.89, 716.91, 716.92, 716.99).
Avascular Necrosis	A diagnosis on a carrier claim over the period of 365-days prior to their index fracture date (ICD-9 codes: 733.40, 733.41, 733.49)
Previous year Medicare spending	Total Part A and B payments made by Medicare for the beneficiary over the period of 365-days prior to their index fracture date.

*Categories of patient race were not defined by study investigators, but instead followed predefined categories used by ResDAC.

© 2020 Floyd SB et al. *JAMA Network Open.*

eAppendix. Instrument Development

The Area Surgery Ratio (ASR) for each local area represents the proclivity of patients with PHF in the area to receive surgery, independent of their demographic and clinical characteristics. Area Surgery Ratios are created by first generating predicted probabilities of surgery for each patient from a multivariate regression model parameterized using the entire patient sample and including all covariates listed in eTable 2. In our models, we used logistic regression to generate predicted probabilities of treatment, equal to

$$(1) \quad \Pr(T)_i = \Pr(T_i = 1|X_l) = \frac{1}{e^{-\hat{\beta}X_{l_i}}}.$$

T_i in equation 1 is an indicator for whether each patient received surgery, X_l is a vector of variables that are controlled for in the regression model, and $\hat{\beta}$ is the vector of parameter estimates produced by the regression.

The ASR for each HRR is then calculated as the ratio of the number of observed to the number of predicted patients that received initial surgery in the area. The ASR for each area, j, will be calculated as

$$(2) \quad ASR_j = \frac{\sum_{i=1}^{N_j} T_i}{\sum_{i=1}^{N_j} \Pr(T)_i}$$

where N_j is the total number of patients (i) in each local area (j), T_i takes a value of 1 if the patient received surgery and 0 otherwise, and $\Pr(T)_i$ is each patients predicted probability of treatment.

© 2020 Floyd SB et al. *JAMA Network Open.*
